# On the use of Action Units and fuzzy explanatory models for facial expression recognition

**DOI:** 10.1371/journal.pone.0223563

**Published:** 2019-10-15

**Authors:** E. Morales-Vargas, C. A. Reyes-García, Hayde Peregrina-Barreto

**Affiliations:** Instituto Nacional de Astrofisica, Optica y Electronica, Luis Enrique Erro 1, Santa Maria Tonantzintla, 72840 Puebla, Mexico; Ulm University, GERMANY

## Abstract

Facial expression recognition is related to the automatic identification of affective states of a subject by computational means. Facial expression recognition is used for many applications, such as security, human-computer interaction, driver safety, and health care. Although many works aim to tackle the problem of facial expression recognition, and the discriminative power may be acceptable, current solutions have limited explicative power, which is insufficient for certain applications, such as facial rehabilitation. Our aim is to alleviate the current limited explicative power by exploiting explainable fuzzy models over sequences of frontal face images. The proposed model uses appearance features to describe facial expressions in terms of facial movements, giving a detailed explanation of what movements are in the face, and why the model is making a decision. The model architecture was selected to keep the semantic meaning of the found facial movements. The proposed model can discriminate between the seven basic facial expressions, obtaining an average accuracy of 90.8±14%, with a maximum value of 92.9±28%.

## Introduction

Facial expressions simplify communicating emotions [1]. In Computer Sciences, Facial Expression Recognition (FER) refers to the identification of emotions in images or video sequences of human faces by computational algorithms. FER is important because of the applications it has in different domains, such as security, affective computing, sociology [2], and facial rehabilitation [3–5]. These applications include human-computer-interaction [6], driver safety [7], human health care [8], and rehabilitation [9–11].

Many works have been reported to tackle FER. These can be split into static and dynamic approaches. Static approaches are based on local descriptors of a frontal face image in which a facial expression is encoded. Dynamic approaches estimate differences between the face in a neutral state and the facial changes in a sequence of frontal images. Given these inputs, either a single image or an image sequence, facial expression recognition systems often proceed by extracting features from the input image set to feed a subsequent classifier that outputs the inferred facial expression [12–15].

Even so, static approaches can get more discriminative power. We prefer dynamic approaches over static ones since these allow the possibility of determining which movements are present in the input sequence. Determining the movements present in the input sequence can lead not only to affording an output label but also to accompanying it with the procedural mechanics of why the model is making a decision.

The necessity of a simple and interpretable model that can also provide procedural mechanics to facial expression recognition guided us to the use of fuzzy models. Fuzzy models are proposed to handle the ambiguity of the linguistics models, such as the facial action coding system, which describes each basic facial expression with small facial movements called Action Units. Actual FER systems can be improved by describing each facial expression not only with its appropriate label, but also with annotations of which facial movements are present in the face. A more exhaustive explanation of facial expressions in an image can be used in medical environments in conjunction with fuzzy models to enlarge FER applications.

The main goal of this research is to model facial expressions through automatically generated fuzzy rules defined over Action Units of the facial action coding system, maintaining a high discriminative power between facial expressions whilst concomitantly explaining why the model is making a decision.

## Methodology

We propose a methodology for FER that is divided into three steps: (i) facial landmark alignment, (ii) feature extraction with pooling, and (iii) classification. The database used provides facial landmarks obtained through Active Appearance Models (AAMs) to track the face. These landmarks are subsequently used to extract the features. Finally, a set of models for each Action Unit (AU) is trained in order to generate rules using fuzzy models to infer the facial expression present in the image sequence.

Each facial movement is called an Action Unit, and it describes the smallest visually discriminable facial deformation for each anatomical facial structure [16]. The AUs with the Facial Action Coding System (FACS) specify 9 upper face movements and 18 lower face movements listed in Table 1.

**Table 1:** Action Units and their corresponding movements.

AU	Movement	AU	Movement
0	Neutral state	14	Dimpler
1	Inner brow raiser	15	Lip corner depressor
2	Outer brow raiser	16	Lower lip depressor
4	Brow lowerer	17	Chin raiser
5	Upper lid raiser	18	Lip puckerer
6	Cheek raiser	20	Lip stretcher
7	Lid tightener	22	Lip funneler
9	Nose wrinkler	23	Lip tightener
10	Upper lip raiser	24	Lips pressor
11	Nasolabial deepener	25	Lips parted
12	Lip corner puller	26	Jaw drop
13	Cheek puffer	27	Mouth stretch

**Fig 1:**
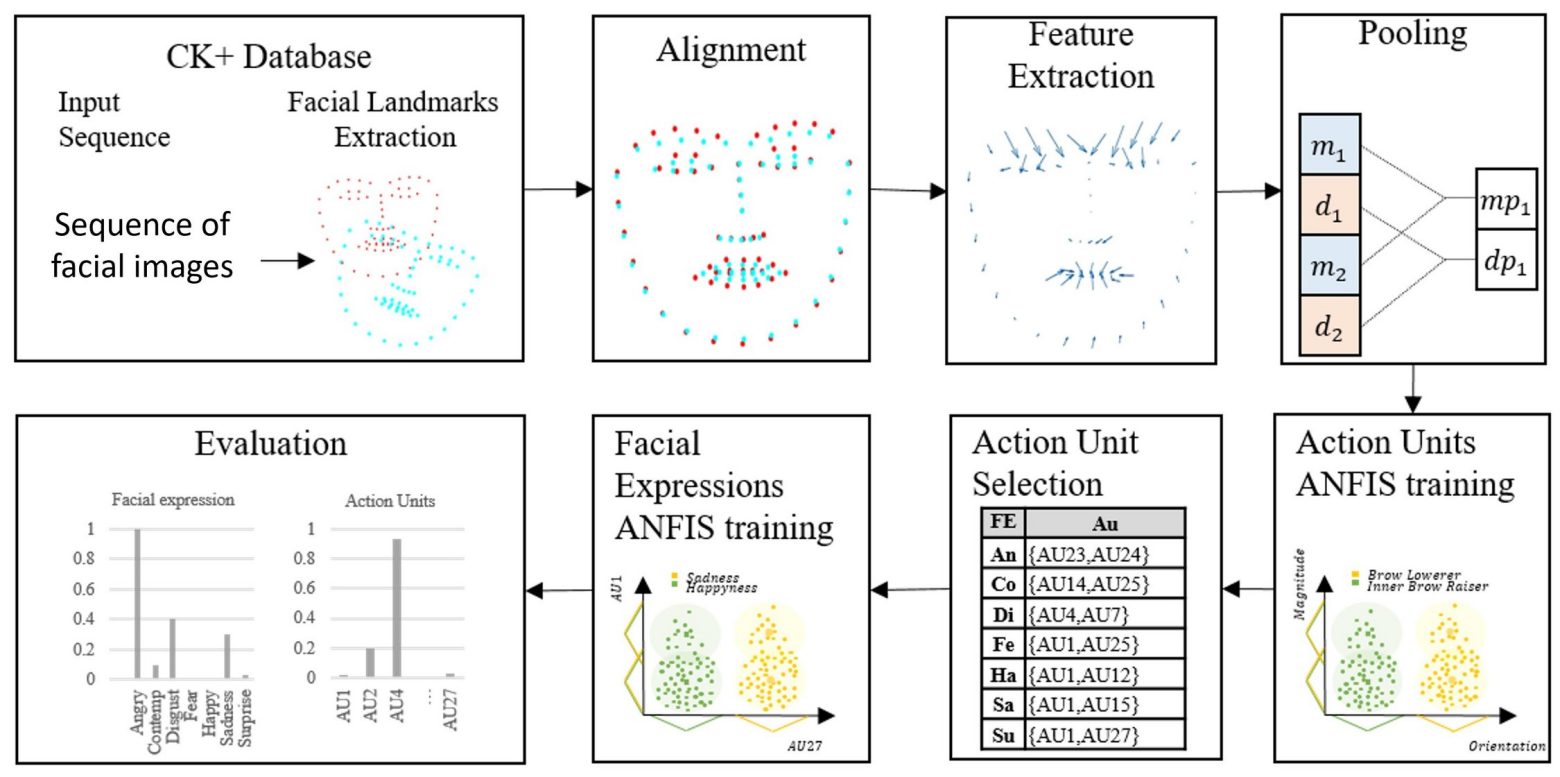
Proposed method for facial expression recognition using fuzzy rules over facial movements.

By using FACS, human experts can detect and encode basic emotions by observing the present AU in the face [17]. Based on the FACS, we trained models to identify each AU. The models are then used to generate rules over the AUs to provide an explainable model that encodes each facial expression into facial movements. AUs related to facial expressions are shown in Table 1 [16]. Fig 1 depicts the proposed Fuzzy Rules Over Action Units for Facial Expression Recognition (FROAUFER) methodology.

### (i) Facial landmark alignment

A set of 68 coordinate pairs *st* = {{*x*_1_, *y*_1_, *x*_2_, *y*_2_, …, *x*_68_, *y*_68_}, *t*} describing the facial shape is the input for our method. Each image *I* of the image sequence *is* = {*I*, *t*} must have its coordinate pair. To avoid orientation, size, and rotation noise between frames, a method to compare two sets of facial landmarks (*st*_*t* = 0_ with *st*_*t* ≠ 0_) is required. Procrustes Analysis (PA) [7, 18] superimposes shapes by optimally translating, rotating, and uniformly scaling objects. PA mitigates geometric distortions in images or landmarks in terms of affine transformations. However, PA is highly sensitive to noise, geometric distortions, and outlier values. When a facial expression is represented, a geometric distortion usually happens. Thus, PA might not be the best choice to align facial landmarks.

In this work, facial landmarks are aligned to reduce the noise in the system using a heuristic method based on affine transformations, ensuring invariance to scale, orientation, and translation. To align facial landmarks, shape values *st* are first normalized between [0, 1]. A correction is then performed to maintain the deformations caused by facial movement. There are two variants to perform the proposed method. The first variation of the facial landmark alignment method consists of a size variation correction in terms of the neutral state. In the first case, which is the Alignment Maintaining the Aspect Ratio (AMAR), the max size of the neutral state is used in all landmarks to perform the normalization between [0, 1] instead of the individual size of each landmark set for a time *t* of the landmarks set *st*. The second variation, the Alignment In Terms of the Neutral State (AITNS), consists in obtaining and using the aspect ratio of the face to normalize the landmarks where $ar=\frac{vms}{hms}$, *vms* being the vertical size of the face, and *hms* the horizontal size of the face.

### (ii) Feature extraction

The performance of a predictive model often depends on the chosen representation [19]. Choosing an appropriate representation is relevant to boost classification rates [20, 21]. The proposed representation comprises three steps: (i) magnitude and orientation computing, (ii) facial size computing, and (iii) pooling.

First, the displacement between the landmarks of the neutral state and the facial expression are obtained. Using the displacements of facial landmarks *dx*_*i*_ and *dy*_*i*_ the magnitude $m_{i}=\sqrt{{\left(dx_{i}\right)}^{2}+{\left(dy_{i}\right)}^{2})}$ and the orientation $d_{i}={\text{tan}}^{-1}\left(\frac{dx_{i}}{dy_{i}}\right)$ for each facial landmark *i* are obtained. Then, a triangulated shape of facial landmarks obtained from [21] is used to compute the size of each anatomical facial area (eyes, mouth, nose, eyelashes, eyelids and chin). Finally, the magnitude, the orientation, and the areas are concatenated to obtain a raw 243-dimensional feature vector. The feature vector is subsequently pooled to obtain a compact representation.

To reduce the number of possible fuzzy rules (243!) and in order not to lose the ability to explain why the fuzzy model is deciding, a dimensionality reduction is necessary. A dimensionality reduction impacts the fuzzy model, generating fewer interpretable rules. Here, a classical exploration of the data was made. In order not to lose the semantic meaning of each AU, distinctive areas of the face were chosen, and the features of each subset were averaged pooled [22]. Using the pooling scheme, the dimensionality was reduced from 243 to 22. Two pooling operations were taken into account to embed the features due to their simplicity, namely average pooling $fa=\frac{1}{p}\sum_{i=1}^{p}v_{i}$ and the max pooling *fm* = *max*(*v*_*i*_), where *i* is the i-th described distinctive area, and *v*_*i*_ is a subset of facial landmarks related to the facial area being embedded.

### (iii) Classification

To classify, we use a granular fuzzy model. This kind of model represents the information as hyperboxes. A hyperbox is commonly an oval region of the decision space [23]. For simplicity, we used a Takagi-Sugeno fuzzy inference system to assign a degree of membership to each AU present in the image.

Each AU is associated to a distinctive area. This association is used to select only those features of the representation related to the AU. For each AU, a Takagi-Sugeno model is generated using a rule generation algorithm [24]. The rule generation algorithm consists of two steps: (i) hyperbox generation, and (ii) rule generation. For the hyperbox generation, subtractive clustering [25] is used with a Gaussian membership function. The fuzzy rule generation algorithm is used to generate an approximation of the model. After that, a tuning step is performed using the Adaptive Neuro-Fuzzy Inference System (ANFIS) training algorithm [26].

Finally, a semantic meaning is assigned to each fuzzy rule obtained. The semantic meaning assigned to each rule helps to infer why the model is making a decision. The partitioned membership values in the interval [0, 1] are partitioned; for each partition, a meaning is assigned (very weak, weak, medium, strong and very strong presence). The inferred membership values of the AU are later used to generate a new representation which contains each AU activated in the image sequence. Before the rule generation, a sequential feature selection for each AU was used to generate a more specific model. Using the selected AUs, a model is generated for each facial expression.

**Fig 2:**
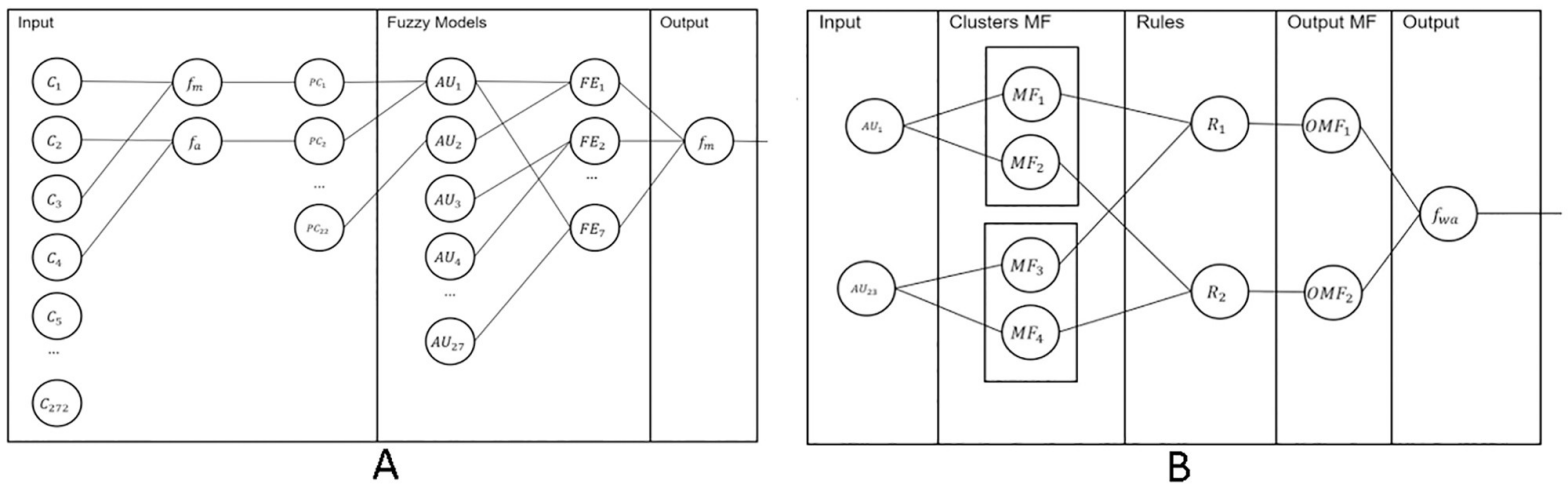
Neuro-Fuzzy representation of the proposed model for facial expression recognition. A) Full model overview and B) overview of the neuron *E*_1_ in the full model.

In general terms, the proposed model can be seen in a Neuro-Fuzzy representation, Fig 2A. In the input, the 243 feature representation is pooled using a pooling function *pf* to obtain the new pooled representation *pr*, which is used as input to the fuzzy set of inference systems for each AU. The results of the AU models are then used to infer which facial expression is observed in an image sequence. On the other hand, in Fig 2B a more detailed explanation of the behavior of the *E*_1_ neuron can be seen (anger). In the input part, the two selected AUs (inner brow raiser and lip tightener) are the input to infer if the facial expression is present or not in the image sequence.

## Data

Two public datasets validated using the FACS for facial expression recognition were used to perform the experiments of this work: The Cohn-Kanade (CK+) dataset and the Radboud Faces Database, which are going to be briefly described in the following sections.

### Cohn-Kanade dataset

The CK+ was obtained from [21]. It consists of 327 validated image sequences from 123 healthy subjects, in which one of the seven basic facial expressions is represented: anger, contempt, disgust, fear, happiness, sadness or surprise. CK+ is labelled by expert judges according to the FACS [27], and has become one of the most widely used for algorithm development and evaluation. The number of samples contained in the CK+ dataset for each facial expression is shown in Table 2.

**Table 2:** Frequency for each facial expression from the CK+ dataset.

Emotion	N
Angry (En)	45
Disgust (Dis)	59
Contempt (Con)	18
Happiness (Hap)	69
Fear (Fe)	25
Sadness (Sad)	28
Surprise (Sur)	83

Each sequence begins with a subject in the neutral state and ends in a facial expression representation. An expert judge manually encoded each sequence using the facial action coding system [16] and labeled which AUs are present in the sequence and their intensities. Coding was done in the following form *label* = {[*au*_1_, *i*_1_], [*au*_2_, *i*_2_], …, [*au*_*n*_, *i*_*n*_]}, where *au* is the number of AUs present in the sequence, and *i* denotes the intensity of the AU. An example of the label is presented in Table 3.

**Table 3:** Label example of a sample in the CK+ dataset. Each sequence in the dataset contains a similar one.

Subject	Sequence	AU labels	Emotion label
01	04	{[4, 0], [7, 5], [17, 4], [23, 4], [24, 4]}	Angry

Using the labels contained in the CK+ dataset, a set of AUs related to each facial expression was selected, and rules for each one were presented (See Table 4). The rules obtained from the selection of the AUs were used to label each sequence with the represented facial expression in the sequence.

**Table 4:** Emotion description in terms of Action Units.

Emotion	Action Units
Angry (An)	AU23 + AU24 must be present
Disgust (Dis)	AU9 o AU10 must be present
Contempt (Con)	AU14 must be present, unilateral or bilateral
Happiness (Hap)	AU12 must be present
Fear (Fe)	AU1+2+4 present, unless AU5 is E, then AU4 can be absent
Sadness (Sad)	AU 1 + 4 + 15, with exception of AU6 + 15
Surprise (Sur)	AU1+2 or 5 must be present, and AU5 should not be stronger than B

Using Active Appearance Models [28], a set of coordinates to describe the face shape in a set of *n* coordinates was obtained. Each coordinate belongs to a vertex of the triangulated shape that describes the face. Each image sequence in the dataset has its own landmarks set.

### Radboud Faces Database

The Radboud Faces Database (RafD) is a set of pictures of 67 models, in which there were Caucasian adults and children (both male and female), as well as Moroccan and Dutch adult males, displaying 8 facial expressions, including the neutral state. The database was obtained from [29]. Five camera angles were used to obtain the images: 180°, 135°, 90°, 45° and 0°. All the images show a model acting one of the basic facial expressions based on the description of the FACS [27] (Table 4). Only the images with 90° were used. For validation of the Radboud faces database, 276 students from the Radboud University participated. We used the frontal images or selected the depicted facial expression and their intensity (from weak to strong), the clarity of the expression (unclear to clear), the genuineness of the expression (faked to genuine) and the valence of the image (negative to positive). Finally, a label for each image was selected using separate analyses of variance (ANOVAs) in the overall agreement. The sample number contained in Radboud database is shown in Table 5.

**Table 5:** Frequency for each facial expression from the Radboud dataset.

Emotion	N
Angry (En)	201
Disgust (Dis)	201
Contempt (Con)	201
Happiness (Hap)	201
Fear (Fe)	201
Sadness (Sad)	201
Surprise (Sur)	201

## Experiments and results

Most training algorithms or methodologies have some settings or parameters from which the user needs to choose. In the case of our model, the three parameters defined for each step of our methodology were the alignment method, the pooling operation (average or max pooling), and clustering type Subtractive Clustering, with the cluster influence or influence radius. The alignment method consists in aligning without taking the neutral state as reference. Instead, it maintains the facial aspect ratio or Procrustes superimposition [21]. Influence radius for subtractive clustering values were between [0.2,0.8]. A 0.1 value for the cluster influence of the subtractive clustering algorithm was discarded due to the high computational cost involved in training the models, and a value greater than 0.8 was not considered for simplicity. The simplest approach for model selection was to run the proposed methodology with the same training data for each combination of parameters. The method was evaluated using leave-one-out replication, allowing the use of the same training/validation data for each combination.

Model selected parameters were the ones that got the highest accuracy (ANOVA: p< 0.05). AITNS was used in the alignment, whereas the average operation was selected in the pooling. We conducted experiments for the radius of influence *γ* of the subtractive clustering algorithm for the AU and facial expression models in the range *γ*_*au*_ = *γ*_*ex*_ = [0.2, 0.8], with steps of 0.1, getting a mean of 90.8±14% for all the parameters. In Table 6, the confusion matrix for the selected model is shown. In the case of the selected parameters, happiness, surprise, and disgust obtained the highest precision. Anger obtained a precision of 0.84, and it was often confused with contempt, disgust and sadness. The confusion can be due to the fact that the aforementioned facial expressions share some Action Units, which make it more difficult for the model to discriminate between them. The model focused on the compression or deformation of the mouth. Contempt got the lowest precision (0.74), and it was frequently confused with sadness, probably because a bilateral dimple causes a similar deformation of landmarks to one caused when a lip corner depressor is found.

**Table 6:** Confusion matrix of facial expression recognition for *γ*_*au*_ = 0.4, *γ*_*ex*_ = 0.2.

	**An**	**Con**	**Dis**	**Fe**	**Hap**	**Sad**	**Sur**
**An**	**0.84**	0.54	0.06	0.00	0.04	0.02	0.00
**Con**	0.01	**0.74**	0.00	0.01	0.07	0.014	0.02
**Dis**	0.02	0.04	**0.92**	0.01	0.00	0.01	0.00
**Fe**	0.00	0.01	0.03	**0.82**	0.06	0.07	0.01
**Hap**	0.00	0.00	0.00	0.01	**0.98**	0.00	0.01
**Sad**	0.06	0.05	0.00	0.06	0.00	**0.83**	0.00
**Sur**	0.00	0.01	0.00	0.01	0.00	0.01	**0.96**

The proposed method was tested in two databases to analyze its behavior. The RafD does not include facial landmarks. To obtain the landmark points, methodologies proposed by Deva-Ramanan (Deva) in [30] and OpenFace [31] were used. For the CK+ database, the Deva-Ramanan facial landmarks were not obtained due to the limitations of the public implementation of the algorithm in terms of the size and color space of the images. The datasets were partitioned into 70% for training and 30% for validation. The obtained results are presented in Table 7.

**Table 7:** Accuracy obtained with the proposed method.

Data	Method	Mean Accuracy
CK+	AAM	0.91±0.03
CK+	Deva	0.76±0.04
RaFD	Deva	0.81±0.02
RaFD	OpenFace	0.45±0.03

### Synthetic model experiments

**Fig 3:**
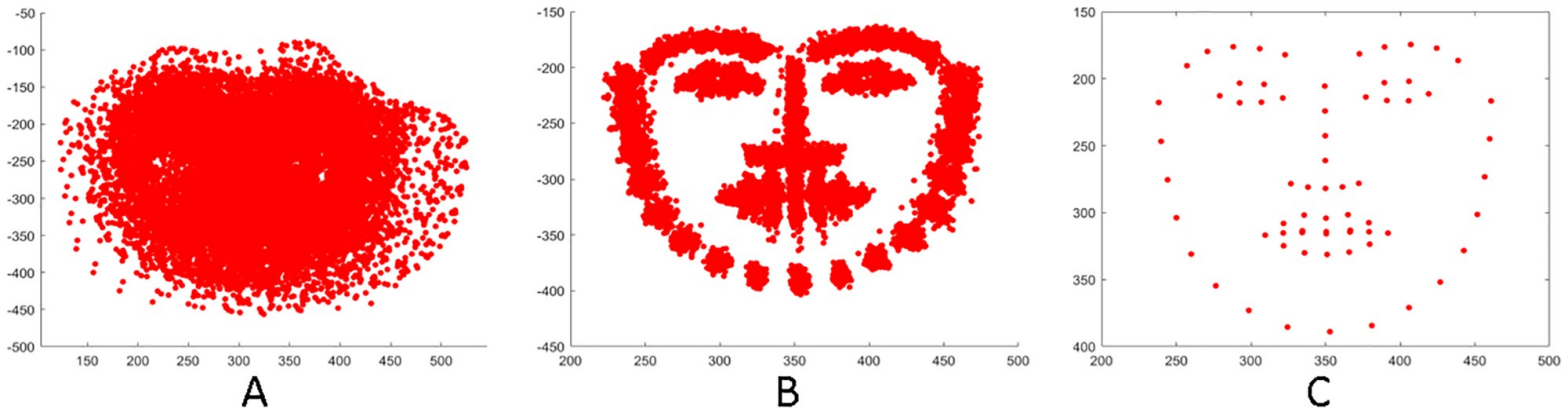
Synthetic generation of a neutral facial expression. A) All samples without PA, B) aligned landmarks after PA, and C) generated model through a mean operation.

One limitation of the model (FROAUFER) is the necessity of a neutral state of the subject to obtain the features. The *AU*0 normalization is the subtraction of the neutral state to a given facial expression. A synthetic model was generated to know the behavior of the model in environments where the neutral state cannot be obtained. The synthetic model was used to perform the AU0 normalization instead of using the neutral state given in the CK+ dataset. From now, the FROAUFER method with the AU0 normalization using the synthetic model will be called FROAUFERAU0. The neutral state model was generated using a random sample of a neutral face obtained from the CK+ dataset. The sample was aligned using PA. PA is well known for its ability to obtain models from a set of landmarks. Remaining samples were fitted to the randomly selected neutral face Fig 3A. A mean operation for each landmark was performed in order to generate the model. An example of the model generated is depicted in Fig 3C. The experiment was performed with the CK+ database. Using a synthetic model, the mean accuracy decreased from 90.8±14% to 70±7%.

### Cross-database facial expression recognition

Facial expression recognition methods have problems obtaining high accuracies when evaluated using the cross-database validation protocol. Even though the environment is controlled within databases (frontal faces, illumination, occlusions, among others), the environment is not controlled across databases [32]. Thus, it is important to know the performance achieved by a model when it is tested over a database different to the used in its training, simulating an enviroment when a system is implemented and a subject is using it. The experiments were organized in the following way. A selected model trained with the CK+ database [21] was used to evaluate each frontal sample in the RaFD database [29], in a leave-one-out replication method with a variation in the clustering influence for the subtractive clustering algorithm *γ*_*exp*_ between [0.2,0.8]. The obtained accuracy for the proposed FROAUFER model for this experiment was of 0.69±0.01, and it is compared with other methods of the literature in Table 8. For the comparison of the accuracy obtained, the selected works are those that use the RaFD for validation purposes. The difference in accuracy obtained among databases can be because of the methods used to validate the facial expressions depicted. For example, JAFFE is not validated using the FACS, but RaFD was. We can conclude that in the case when the system needs to be used in a real application, the facial expressions to be detected should resemble those described using the FACS to improve detection rates.

**Table 8:** Comparison of the accuracy obtained in a cross-database process.

Method	Train	Target	Accuracy
[32]	6 databases*	RaFD	0.85±0.04
[33]	JAFFE	RaFD	0.52±N/R
[33]	TFEID	RaFD	0.55±N/R
FROAUFER	CK+	RaFD	0.69±0.01

*CK+, MMI, RaFD, KDEF, BU3DFE, ARFace

## Discussion

Considerable effort has been made to tackle facial expression recognition, but current solutions have favored discriminative over explicative power. Explicative power does not aim to obtain the highest accuracy; instead, it gives additional information to the user to perform a better understanding of what is represented in an image sequence. Let us suppose that an assist system for rehabilitation of subjects of facial paralysis is wanted. A more reliable output for a system will be the grade of paralysis and affected area, rather than only if the subject is suffering or not from the disease.

The results obtained with the proposed method are similar to recent works that have used a dynamic approach or facial landmarks for this task and also providing a more detailed description of the movements present in the image sequence (Tables 8 and 9). Dynamic approaches, such as CAPP and S+C, begin with a normalization step using procrustes analysis, which is later normalized by substracting the neutral face from the facial expression, obtaining an accuracy of 77±2.9% for their models, with a maximum of 88% (Table 9). On the other hand, [15] proposed a descriptor which captures the changes of 560 angles obtained with the combination facial landmarks. This descriptor can have three values: -1 when the angle decreases, 0 when the angle does not move, and 1 when the angle increases. It is also invariant to pose; for this reason, a normalization or alignment is not required. Although some initial steps to take advantage of the diffuse models [19][3] have been presented, a limited number of actions are used without taking into account the validation provided by the FACS.

**Table 9:** Comparison with related works that use dynamic approaches.

Work	Data	Method	Interpretable	FACS	Mean Accuracy
[21]	CK+	CAPP	no	no	0.80±N/R
[21]	CK+	S+C	no	no	0.88±N/R
[15]	CK+	ORB	no	no	0.92±N/R
[3]	JAFFE	MFS	14 rules	no	0.87±N/R
[19]	JAFFE	FRM	565 rules	no	0.96±N/R
[30]	CK+	RM	no	no	0.85±N/R
[34]	JAFFE	FKC & SVMs	no	no	0.97±N/R
Proposed	CK+	FROAUFERAU0	yes	yes	0.70±0.07
Proposed	CK+	FROAUFER	yes	yes	0.91±0.03
Proposed	RaFD	FROAUFERAU0	yes	yes	0.61±0.05
Proposed	RaFD	FROAUFER	yes	yes	0.81±0.61

The model presented in this work contains a few rules due to the method used to select the rules, with major impact in the accuracy of the classifier. The proposed model used a system that explains the appearance of the face through models using the smallest distinguishable facial movements. This is the greatest strength of the proposed methodology.

**Fig 4:**
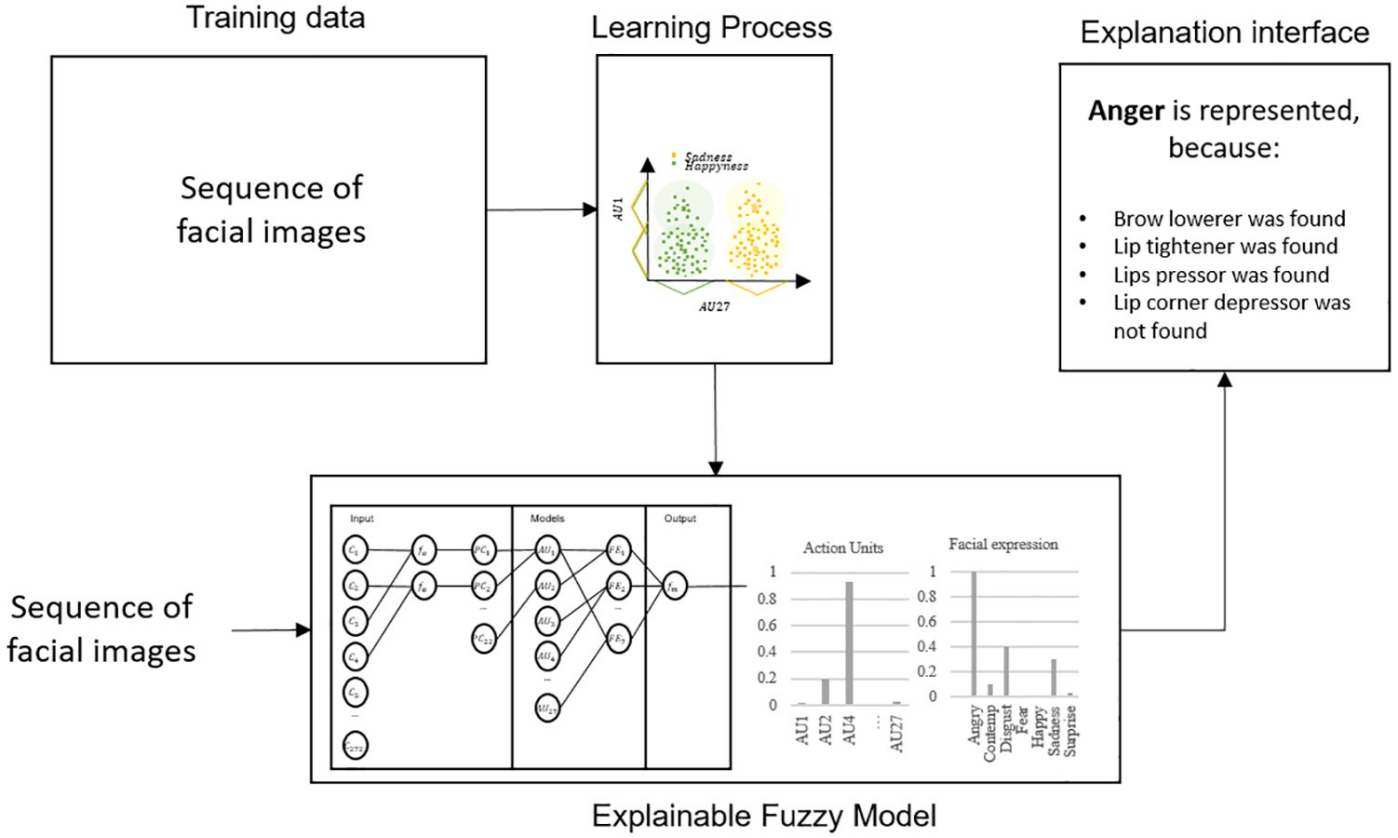
Fuzzy model for facial expression recognition which can lead to an explanation interface.

The FROAUFER methodology starts with a heuristic based on affine transformations, rather than with a Procrustes analysis [21]. In FROAUFER, the use of the heuristics based on affine transformations for facial landmarks alignment improved significantly the accuracy over Procrustes superimposition or without aligning the facial landmarks. The 22-dimensional representation is smaller than the others presented in the literature [15, 33, 35], reducing the time for feature extraction, and obtaining a mean accuracy of 90.8±14, with a maximum of 92.9±28. The main feature that stands out is that the results are interpretable, which can generate an interface explaining why the model is making a decision (Fig 4). Experiments were carried out in order to know the effects of the use of a synthetic model to avoid the dependency on the subject’s neutral state. Experiments have shown that our model can obtain accuracies greater than 0.70 without calibration, which is an initial step to alleviate the necessity of a starting neutral state.

## Conclusions

In this work, we presented the design, implementation, and experiments carried out with a simple model for recognition of facial based on facial movements of distinctive areas. The presented model improved fuzzy models, which explain the appearance of the face through rules. The proposed model differs from the models presented in the state of the art in its ability to explain why it is making a decision. It uses an appearance system which describes facial expressions in terms of movements of facial distinctive areas, giving a more detailed explanation of what movements are in the face, and why it is making a decision. It also keeps the semantic meaning of the facial action coding system.

As an attempt to expand the method to another database, a set of landmarks was extracted using two methods: Deva and OpenFace. Obtained results suggest that detailed training and parameter selection can improve the performance of the model. It is important to point out that the results suggest that the facial landmarks selection method had a direct impact on the model efficiency.

A synthetic model obtained using the training data was generated as an attempt to mitigate the necessity of the model to have a neutral state to perform the AU0 normalization. As expected, in most cases the accuracy was lower than the one obtained using the real neutral state of the subject.

A method for decoding and explaining facial expressions in terms of Action Units using fuzzy logic was proposed. Fuzzy decision rules were generated through subtractive clustering of facial landmarks. Movement descriptors and an assignment of semantic meaning to the rules provide the explanatory mechanism. We obtained an average accuracy of 90.8 ± 14%, with the maximum being 92.9 ± 28% when *γ*_*au*_ = 0.4, *γ*_*ex*_ = 0.2, parameters of the clustering algorithm. Although the clustering results for the parameters *γ*_*au*_ and *γ*_*ex*_ are similar for values greater than 0.2 (ANOVA: p< 0.05), the recognition rate for a specific facial expression can vary. Modeling each facial expression with a cluster influence for each one, *γ*_*au*_ and *γ*_*ex*_, can probably improve the hit rate for facial expression.

## Future work

After analyzing the obtained results, several issues of the proposed methodology could be improved and considered for proposals of future work. These proposals include the following. If facial landmarks are going to be used as a tool to detect facial distinctive areas, a more reliable and generalized method for facial landmarks alignment based on the Procrustes analysis can be developed. A possible way to do this is by extending the analysis to keep distinctive deformations of the shape to be superposed. A better pooling scheme is needed to experiment using various combinations of features. The pooling scheme needs to maintain the captured information by the feature representation. If facial landmarks are not going to be used, a more sophisticated method for facial distinctive areas and movement detection, such as optical flow, can be used, thereby reducing the cost of the location and fitting of the landmarks.
